# Micro- and nano- bentonite to improve the strength of clayey sand as a nano soil-improvement technique

**DOI:** 10.1038/s41598-023-37936-x

**Published:** 2023-07-05

**Authors:** Mohadeseh Cheraghalikhani, Hamed Niroumand, Lech Balachowski

**Affiliations:** 1 Department of Civil Engineering, Faculty of Engineering, Buein Zahra Technical University, Qazvin, Iran; 2 Department of Geotechnical and Hydraulic Engineering, Faculty of Civil and Environmental Engineering, Gdansk University of Technology, Gdansk, Poland

**Keywords:** Environmental sciences, Engineering, Nanoscience and technology

## Abstract

Nano-additives results in the formation of nano-cementation (NC). This process is recently used to improve the durability of various building materials. NC used to improve the strength of untreated soil materials, also known as nano soil-improvement (NSI). In few years, the role of nano-additives in various types of soils were developed. In this research, the role of micro- and nano- size of bentonite as soil stabilizer was evaluated as first few research to improve geotechnical properties of soils. Nano-additives prepared by micro- and nano- sizes of bentonite were blend with four formulations. These formulations of micro- and nano- additives at concentrations of 0, 1, 2, and 3%, namely 0% Micro-Bentonite, 1% Micro-Bentonite, 2% Micro-Bentonite, 3% Micro-Bentonite, 0% Nano-Bentonite, 1% Nano-Bentonite, 2% Nano-Bentonite, and 3% Nano-Bentonite, respectively. These formulations of micro- and nano- additives were separately added to soil. Specimens with 3% nano-bentonite showed significant improvement in unconfined compressive strength (UCS) of soil that was more than 2.3-times higher than control specimen in 7-d curing time. Also the performance of micro-bentonite resulted in improvement in UCS of soil that was more than 1.1-times higher than control specimen at 7-d curing time. The secant modulus at 50% of peak stress (E50) of the samples treated with micro- and nano- additives increased in comparison to untreated specimens. Further, X-ray fluorescence (XRF), scanning electron microscopy, and X-ray diffraction analyses characterized micro- and nano- structures of soil specimens, and showed the performance of nano-additives in improving strength of soils. Results show that nano-bentonite as a type of nano-additives is an effective means of increasing the strength of soils. This research shows the significant of nano-bentonite in soil improvement, as a NSI technique.

## Introduction

Untreated soils are types of loose, loosely compacted soils that can be unstable and can cause problems for building foundations. Construction on untreated soils can lead to serious consequences, such as cracks in building structures and foundations. Common techniques for improving soils include deep soil mixing, stone columns, jet grouting, pre-loading and related methods, but these techniques may not be sufficient for some types of soils^.^ Using additives can be an effective way to improve soils and make them more stable. Additives can include traditional stabilizers like lime, cement, fly-ash, and related additives. Each of these materials has its own advantages and disadvantages, and it is important to study the differences between them to understand the best options for improving untreated soils^1^.

According to these conditions, researchers are finding new techniques in soil improvement. There are few studies where bentonite showed its potential in increasing the strength of soils^2–25^. Bentonite is one of recent environment friendly additives used to improve the geotechnical properties of soils although strength is limited. Nano-additive (NA) is one of the recent methods used to improve the geotechnical properties of soils. NA is a type of nano soil-improvement (NSI) techniques. Recently, nano-additives (NA) with outstanding mechanical properties has been used for soil stabilization in a few studies^2–25^. He et al.^2^ evaluated the role of modified Na-bentonites in calcareous purple soil. Yu et al.^3^ investigated two types of organic bentonites with heavy metals in soil stabilization. Liu et al.^4^ tested chitosan/bentonite and Cr(VI) composites in soil improvement. Pokharel and Siddiqua^5^ conducted a Canadian case study on the combination of calcium bentonite clay and fly ash in organic soils. A few researchers such as Sun et al.^6^ evaluated the role of cadmium (Cd) and lead (Pb) using bentonite in soil improvement: Bani Baker et al.^7^, Liu^8^, Cheng et al.^9^ and Taha and Taha^10^ developed the role of nano-bentonite in sandy and clayey soils. Kozlov et al.^11^ evaluated the environmental sustainability performance of micro-bentonite. The composition of cement and bentonite was controlled by Li and Zhang^12^, Bellil et al.^13^, Estabragh et al.^14^, and Consoli et al.^19^. Hussein and Ali^15^ did a research on the role of polypropylene fiber in expansive soils while Muhammad and Siddiqua^16^ analyzed the feedback of bentonite magnesium-alkalinization at the micro-level in silty sand. El Aal et al. evaluated the role of sodium chloride as a case study in alluvial soils^26^. Muthukkumaran and Selvan^17^ analyzed the combination of montmorillonite-rich bentonite in clay soils. Estabragh et al.^18^ evaluated clay soil with MTBE as a ground improvement method. Falamaki et al.^20^ tested the composition of bentonite and phosphate in clay improvement. The role of microbial performance with bentonite in coarse ground improvement was investigated by Zhao et al.^21^ and Li et al.^27^. A few researchers such as Shourijeh et al.^22^, Firoozfar and Khosroshahiri^23^, and Cheng et al.^9^ used different micro- and nano-sized clay types in erosion, landfill, and consolidation improvement. The importance of nano-additives in soil stabilization was described in existing research^24,25^. Sakr et al.^28^ evaluated the role of rice husk powder as a case study in swelling soil.

According to a literature review, no research has been conducted on the preparation of nano-additives without the contribution of chemicals using lower cost and energy by mechanical methods. The most studies have been used powder particles in soil stabilization^2–25,27,28^. The lack of research on the effects of the powder base of micro- and nano- additives on soils led this study to examine the effect of soil stabilization by micro-bentonite and nano-bentonite using the suspension method. Bentonite is a natural clay which is widely used in many fields, particularly in building and public works. It is used as an adjuvant in the manufacture of concrete, as a binder for soils, as a waterproofing agent for foundations, and as a containment material for nuclear waste. The advantages of bentonite include its abundant availability, low cost, ability to retain water, and ability to improve the physical and chemical properties of materials to which it is added. This study was conducted to stabilize and improve the soil properties, considering the economic justification and optimal use of the bentonite micro- and nano- particles, which had not been done in clayey sand soil previously. No previous research has prepared nanoparticles without chemicals using low-cost and energy-efficient mechanical methods, according to a literature review. This research stabilized and improved the soil properties with soluble nanoparticles, considering the economic justification and optimal use of bentonite nanoparticles. This has not been done before, to the best of our knowledge. Comparing the effects of micro-bentonite and nano-bentonite is an important research topic to understand the potential advantages of nano-bentonite over micro-bentonite. Nano-bentonite may have unique properties that may make it more effective at improving clayey sand soils than micro-bentonite but understanding the differences between the two is important to determining the best options for construction projects. This research aims to study the differences between micro-bentonite and nano-bentonite to improve clayey sand soils. The main objective of this research is to compare the effects of micro-bentonite and nano-bentonite as additives on clayey sand soils. This includes the study of the physical and mechanical properties of improved clayey sand soils, as well as their ability to withstand loads and maintain their stability over time. Tests were carried out in the laboratory to understand the differences between micro-bentonite and nano-bentonite, on clayey sand soils.

Thus, in this research, a fixed amount of micro-bentonite and nano-bentonite added from lower to high concentrations at 0, 1, 2, and 3% was used as stabilizer to improve the geotechnical properties of clayey sand soils. All such clayey sand soils were evaluated to obtain Atterberg limits, elastic modules (E50), and unconfined compressive strength (UCS). All specimens were characterized to evaluate potential of micro- and nano- bentonite in the enhancement of performance characteristics of clayey sand soils using SEM, XRD, and XRD analyses. The results of this research could be used to develop new techniques to improve untreated soils and to inform building standards and codes.

## Materials and methods

### Materials

**Figure 1:**
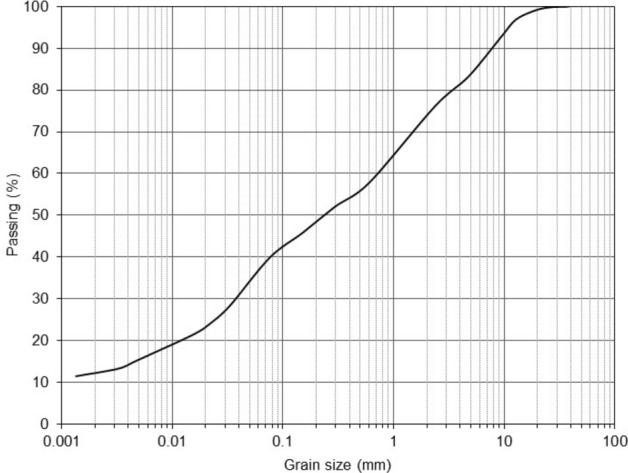
Grain-size distribution plot for the clayey sand.

Clayey sand soil samples were prepared from Survajin Aghigh in Qazvin Province, Iran. The sample soil was classified as clayey sand (SC) according to the unified soil classification system (USCS). The gradation curve, shown in Fig. 1, indicates that sample soil consists of 17% gravel, 43% sand, 40% clay and silt. Table 1 presents the soil properties, such as Atterberg limits, moisture content, and dry unit weight, which were obtained from the soil survey report. Micro-bentonite used in this research is commercially available in Iran. The physicochemical characteristics (XRF analysis) of micro-bentonite used are given in Table 2.

**Table 1:** Index properties of soil.

Parameter	The amount
LL (%)	41
PL (%)	20
PI (%)	21
USCS classification	SC
Optimum moisture content (%)	7.5
Maximum dry unit weight (kN/m^3^)	19.80

**Table 2:** Chemical composition used micro-bentonite (MB).

Chemical compound (Oxide)	Weight content (%)
Na_2_0	1.94
MgO	3.23
Al_2_O_3_	15.08
SiO_2_	51.58
SO_3_	0.74
CL	0.57
K_2_O	1.31
CaO	6.11
TiO_2_	0.47
Fe_2_O_3_	2.97
BaO	2.35
L.O.I	13.65

### Nano-bentonite production

The micro-bentonite was used to produce nano-bentonite. The chemical composition of nano-bentonite is given in Table 3. A ball mill used to produce nano-bentonite powder. The nano-bentonite’s powder was converted into suspension by a homogenizer mixer, termed as nano-bentonite. Nano-bentonite was stored at room temperature before usage.

**Table 3:** Chemical composition used nano-bentonite (NB).

Chemical compound (Oxide)	Weight content (%)
Na_2_0	2.14
MgO	3.05
Al_2_O_3_	14.88
SiO_2_	51.23
SO_3_	0.76
CL	0.39
K_2_O	1.22
CaO	5.94
TiO_2_	0.42
Fe_2_O_3_	2.96
BaO	1.9
L.O.I	15.08

### Sample preparation

**Figure 2:**
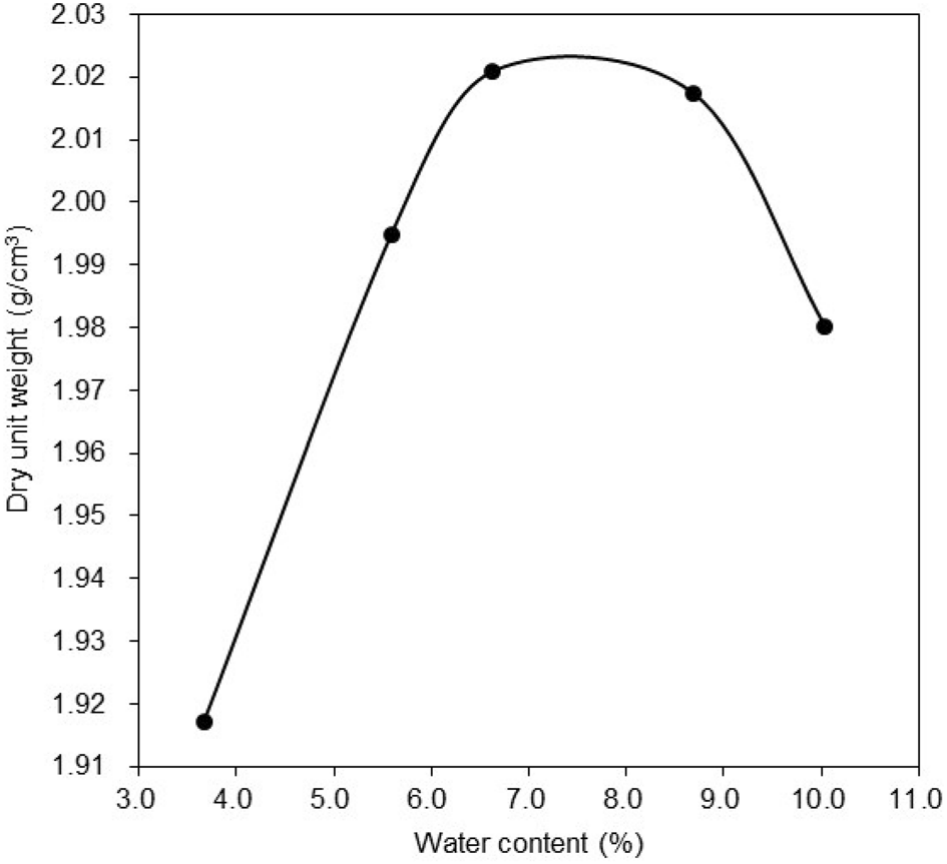
The optimum water content.

The sample of clayey sand was dried in oven at 105 °C. Grain-size distribution plot for the clayey sand is given in Fig. 1. In this research, two types of additives in micro and nano size evaluated in soil mechanics laboratory. In the first type, micro-bentonite with amount of 0, 1, 2, and 3% by dry weight was added into the clayey sand soil and mixed to obtain a homogenous sample. In the second type, nano-bentonite with amount of 0, 1, 2, and 3% by dry weight of soil was introduced into the clayey sand and mixed to obtain a homogenous material. The final clayey sand specimens with 0, 1, 2, and 3% micro- and nano- bentonite are coded as bentonite and nano-bentonite, respectively. The optimum water content to prepare soil samples was evaluated as per standard Proctor compaction test shown in Fig. 2. All the specimens were tested in various curing times such as 1, 7, and 28 days.

The standard compaction test on the graph in Fig. 2 shows the maximum dry density of an untreated clayey sand sample. The standard compaction test of the clayey sand sample shows that the compaction curve increases to 2.02 g/cm^3^ dry unit weight at 7.5% water content. The optimum water content of untreated clayey sand is 7.5%, which corresponds to the maximum dry density of 2.02 g/cm^3^.

### Atterberg limits

The Atterberg limits which determine the plasticity properties of a soil which are important for the valuation of the soil stability. The liquid limit, plastic limit, and plasticity index were determined. These values from clayey sand specimens with micro- and nano- bentonite additives were evaluated according to ASTM D 4318.

### Unconfined compressive strength (UCS)

A series of test specimens, each 50 mm in diameter by 100 mm in height, was prepared at optimum moisture content for UCS determinations at various curing times. These strength values for clayey sand specimens with micro- and nano- bentonite additives were evaluated based on ASTM D 2166. An axial strain rate of 1.27 mm/min was applied. These samples were tested for curing periods of 1d, 7d, and 28d. The secant modulus at 50% of peak stress (E50) of treated micro- and nano- samples was determined and compared to untreated specimen based on stress—strain behavior registered in UCS test.

### XRF, XRD, and SEM analyses

X-Ray Fluorescence (XRF) analysis is an analytical technique that uses X-rays to excite atoms in a sample and measure the light emitted by the excited atoms. This light can be analyzed to determine the chemical composition of the sample. X-ray fluorescence analysis is a fast and accurate way to determine the composition of many types of materials, such as minerals, metal alloys, chemicals and consumer products. It is widely used in the mining, petroleum and metallurgical industries, as well as in environmental and occupational health and safety applications. These values from clayey sand specimens with micro- and nano- bentonites were evaluated based on ASTM E1621-13 in XRF. X-ray diffraction (XRD) testing is a method used to study the crystal structure of a substance. It involves the use of X-rays to produce light diffraction on atoms in a crystal, producing an image that can be analyzed to determine the spatial arrangement of atoms in the crystal structure. The X-ray diffraction test made it possible to determine the crystal structure of bentonite. XRD was evaluated to identify minerals and other crystal structure of soil samples including treated and untreated specimens with micro- and nano- bentonites using X-ray diffractometer in Tehran, Iran. These values from clayey sand soil with micro- and nano- bentonite additives were evaluated based on BS EN 13925-1 in XRD. Scanning Electron Microscopy (SEM) analysis is an imaging technique used to study the structure and composition of materials at the micro and nano scales. It permits using an electron beam to scan the surface of a sample and produce a two-dimensional image of its surface. The microstructures of all gold-coat specimens were evaluated under a Scanning Electron Microscopy (SEM) in Tehran, Iran. These values from clayey sand specimens with micro- and nano- bentonite addives were evaluated based on 5 µ and 500 nm scales in SEM.

## Results and discussion

### Atterberg limits

**Figure 3:**
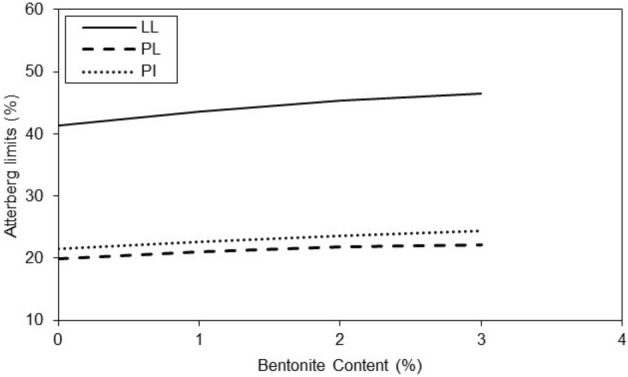
Variations of Atterberg limits of treated clayey sand with micro-bentonite content.

**Figure 4:**
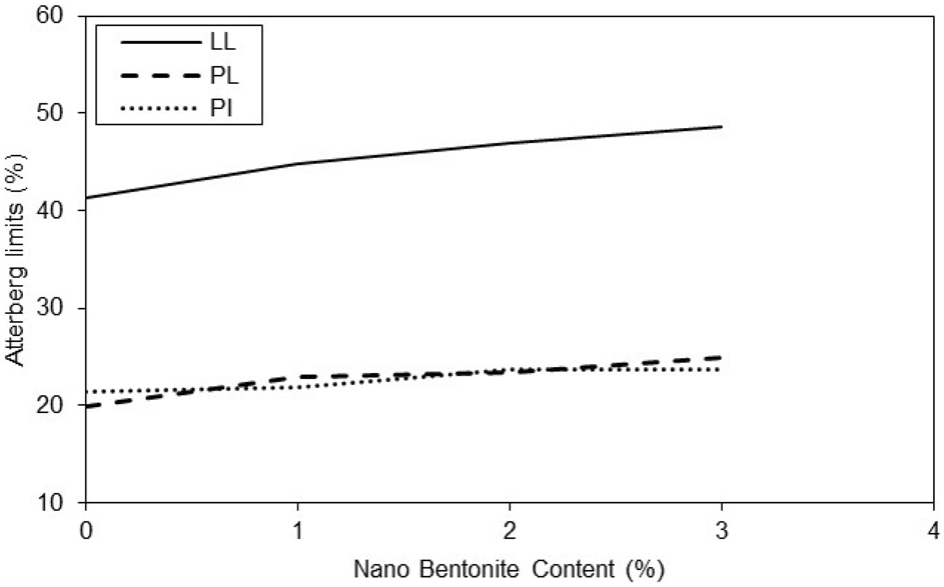
Variations of Atterberg limits of treated clayey sand nano-bentonite content.

Generally the behavior of untreated and treated clayey sand in related to the water content. According to Figs. 3 and 4, the liquid limit values of the clayey sand specimens increased with an increasing amount of micro-bentonite till 3% concentration and similar behavior of plastic limit and plasticity index were observed. In specimens with nano-bentonite, the liquid limit, plastic limit, and plasticity index of the treated specimens increased till 3%micro-bentonite content.

**Figure 5:**
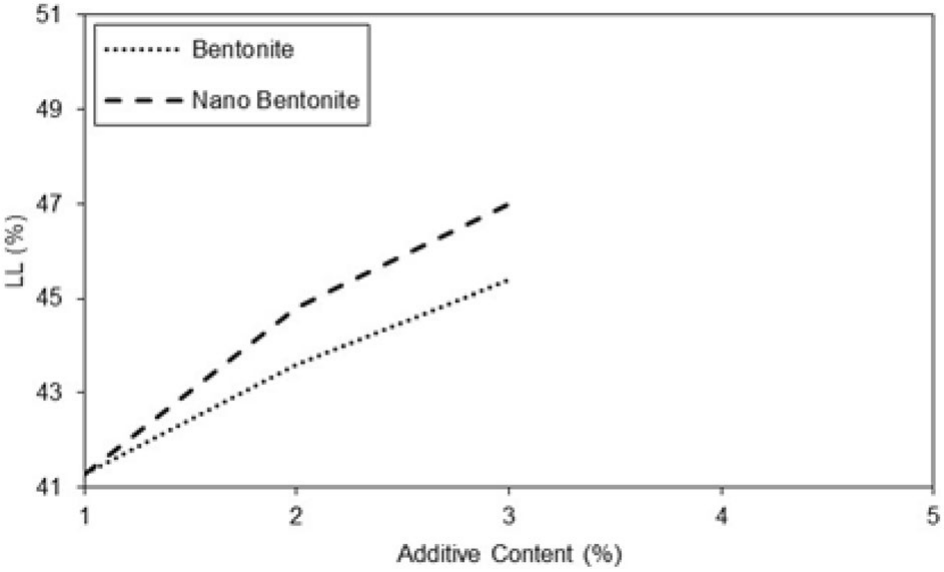
Variations of liquid limit of treated clayey sand with micro- and nano- bentonite content.

**Figure 6:**
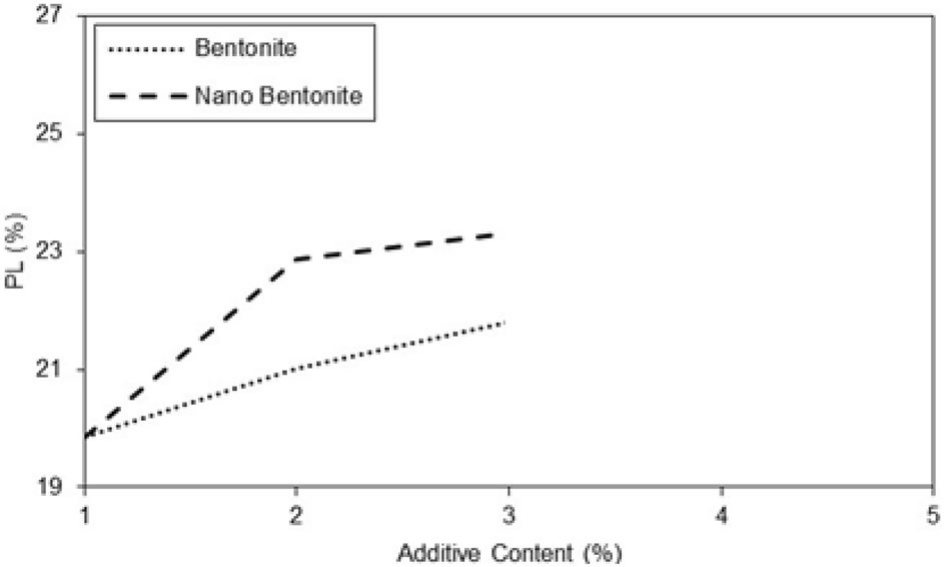
Variations of plastic limit of treated clayey sand with micro- and nano- bentonite content.

The results of Fig. 5 show that the additives have a significant effect on the liquid limit of specimens improved with micro-bentonite and nano-bentonite. In general, the additives increased the liquid limit of micro-bentonite and nano-bentonite. However, the effect was more marked for nano-bentonite. It is observed that with an additive content of 1%, the liquid limit is 41% and with an additive content of 3%, the liquid limit reaches 47%. There is a notable increase in the liquid limit of 6% between the additive content of 1 and 3%. The additive content had a significant effect on the liquid limit of specimens with nano-bentonite, much higher as compared to micro-bentonite additive. In general, the higher additives content the larger plastic limit of specimen improved with micro bentonite. However, it has also been observed that higher additives content can significantly increase the plastic limit of specimen with nano-bentonite. Noticeable peaks at 2 and 3% additive content are observed for the nano-bentonite and a noticeable peak at 3% additive content for the micro-bentonite in Fig. 6. It can be assumed that with an additive content of 3%, the largest plastic limit of nano-bentonite is observed. In conclusion, the results suggest that the micro-bentonite content can exert an influence on the plastic limit of clayey sand, and that nano-bentonite can be considered as a more effective additive to improve the plastic limit of untreated clayey sand due to its increased ability to evenly distribute additives in untreated clayey sandy soil.

**Figure 7:**
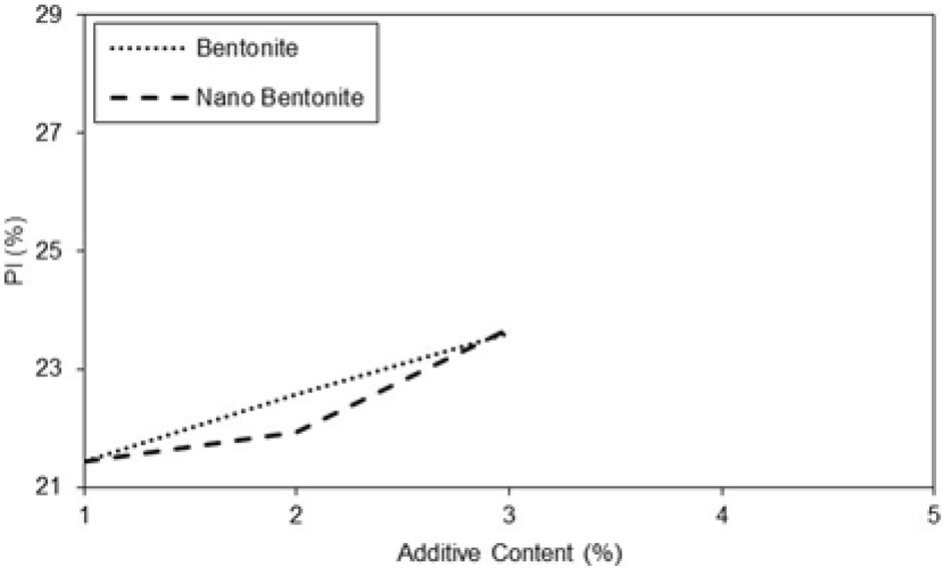
Variations of plasticity index of treated clayey sand with micro- and nano- bentonite content.

In treated soil the plasticity index increases with additive content (Figs. 6 and 7). With an additive content of 2%, the plasticity index of the specimen improved with nano-bentonite is lower than that of micro-bentonite. It was observed at additive content of 3%, the plasticity index of the specimen treated with nano-bentonite is lower than that improved with micro-bentonite. Addition of nano-bentonite with the process of nano-cementation aids flocculation of the clayey sand particles that results in better behavior of these soil samples than the specimens treated with micro-bentonite.

### Unconfined compressive strength (UCS)

**Figure 8:**
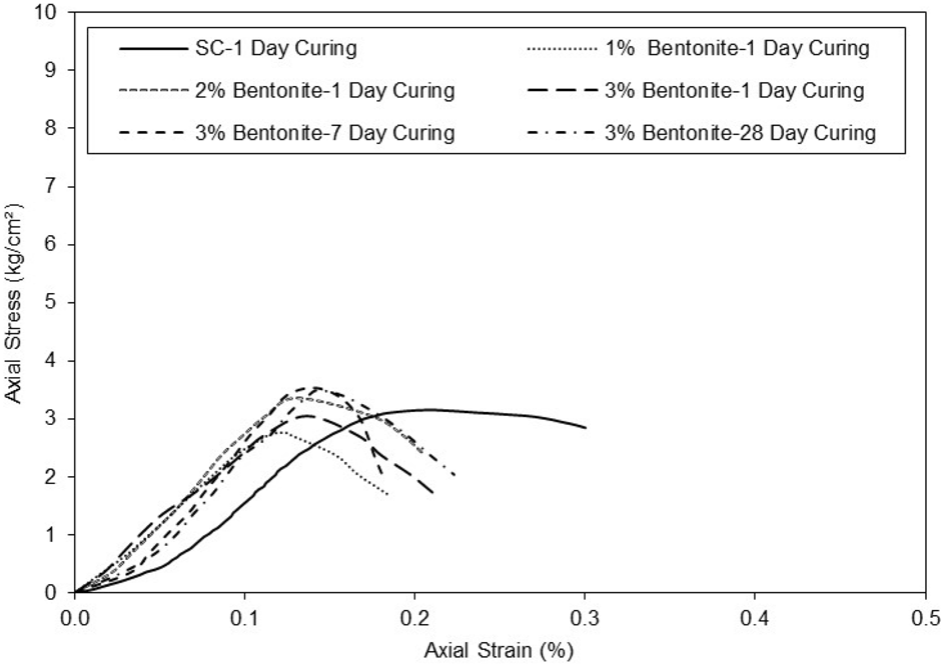
Mobilized shear strength for untreated and treated clayey sand specimen for various percentages of micro-bentonite in different curing periods. Vertical scale.

The results on the graph in Fig. 8 of axial strain versus axial stress showed that the addition of micro-bentonite to clayey sand soil influenced the mechanical properties of the untreated specimens.

The results suggest that the addition of micro-bentonite can be used to strengthen clayey sand and improve their ability to resist deformation and failure under heavy loads. The clayey sand curve at 1d of cure increases gradually until it reaches its maximum with an axial stress of 3 kg/cm^2^ for an axial strain of 0.18%. The treated clayey sandy specimen with the addition of 1% micro-bentonite at 1d of maturation reaches its maximum optimum at an axial stress of 2.8 kg/cm^2^ for an axial strain of 0.11%. Adding 1% micro-bentonite to the clayey sand specimen decreased the axial strain at failure. The clayey sand sample with the addition of 2% micro-bentonite at 1d of maturation reaches its maximum optimum at an axial stress of 3.3 kg/cm^2^ for an axial strain of 0.12%. The clayey sand sample with the addition of 3% micro-bentonite at one day of maturation reaches its maximum optimum at axial stress of 3 kg/cm^2^ at axial strain of 0.13%. Axial strain at failure decreases with the micro-bentonite content. The clayey sand sample with the addition of 3% micro-bentonite at 7d curing times reaches its maximum optimum at an axial stress of 3.5 kg/cm^2^ for axial strain of 0.13%. The clayey sand sample with the addition of 3% bentonite at 28d of maturation reaches its maximum optimum at an axial stress of 3.5 kg/cm^2^ for an axial strain of 0.13%. Adding 3% micro-bentonite to the sample increased the axial stress by 0.5 kg/cm^2^ (3 kg/cm^2^/3 kg/cm^2^) in 7 days curing times. It can be assumed that the addition of 3% micro-bentonite to clayey sand stabilizes the value of axial stress at failure regardless the curing time. However, it can also be assumed that the axial stress and strain at failure stabilize after 7d. There is not much difference between the curve representing the addition of 3% bentonite at 7d of cure and the curve representing the addition of 3% bentonite at 28 days of cure.

**Figure 9:**
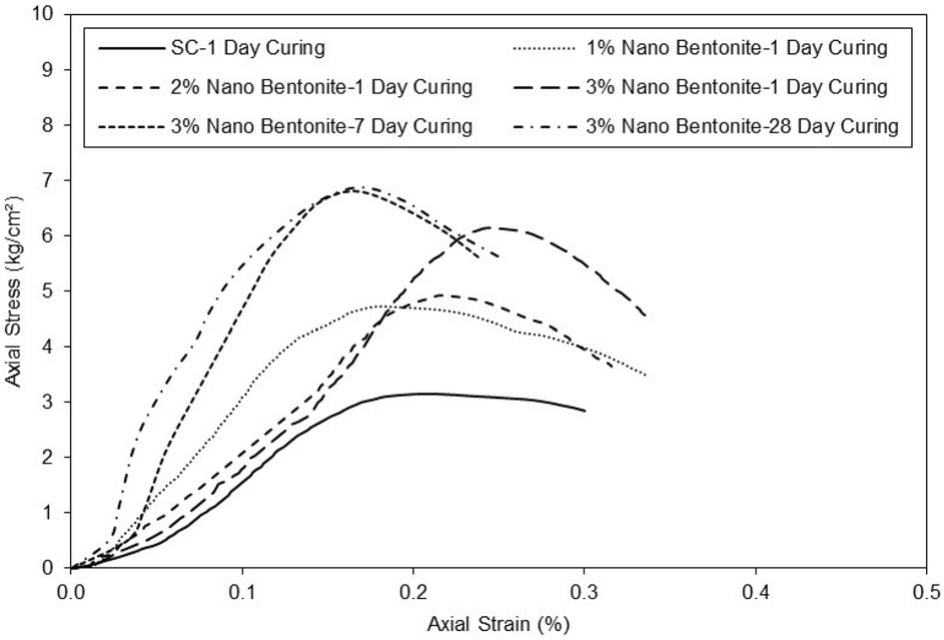
Mobilized shear strength for untreated and treated clayey sand with various percentages of micro-bentonite in different curing periods.

The Fig. 9 shows the axial strain as a function of the axial stress of the clayey sand with the addition of nano-bentonite. The curve for the clayey sand at 1d curing increases progressively until it reaches its maximum with an axial stress of 3 kg/cm^2^ for an axial strain of 0.18%. The clayey sand specimen with the addition of 1% nano-bentonite content at 1 day curing reaches its maximum optimum at axial stress of 4.5 kg/cm^2^ for an axial strain of 0.17%. The clayey sand sample with the addition of 2% nano-bentonite content at 1d curing reaches its maximum optimum at axial stress of 5 kg/cm^2^ for axial strain of 0.21%. The clayey sand sample with the addition of 3% nano-bentonite content at 1 day curing reaches its maximum optimum at axial stress of 6 kg/cm^2^ for axial strain of 0.25%. The higher additive content the larger axial stress at failure.

**Figure 10:**
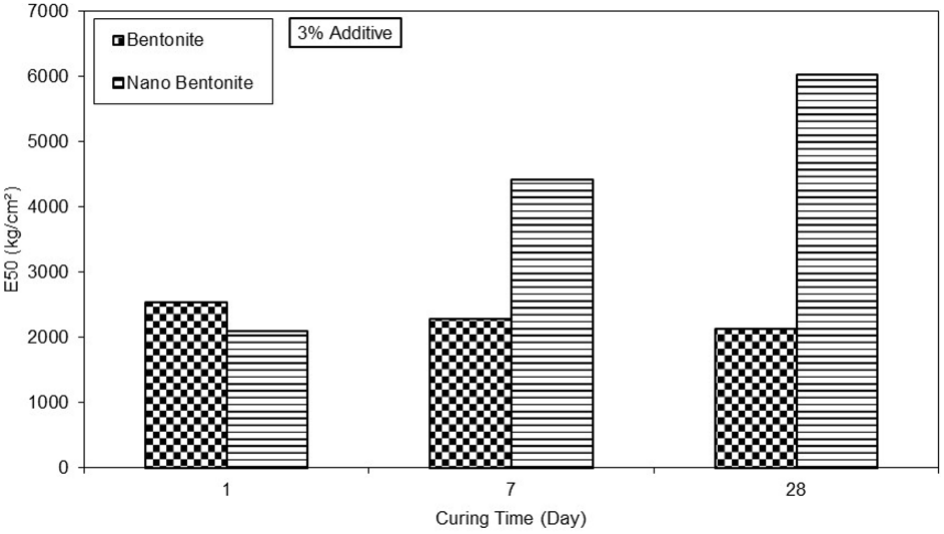
E50 versus various curing times for treated clayey sand with 3% content of micro- and nano- bentonite.

The clayey sand sample with the addition of 3% nano-bentonite content at 7d curing reaches its maximum optimum at axial stress of 7 kg/cm^2^ for axial strain of 0.15%. The clayey sand sample with the addition of 3% nano-bentonite at 28d curing reaches its maximum optimum at axial stress of 7 kg/cm^2^ for axial strain of 0.15%. It can be seen again that at 7d curing, the strength of clayey sand sample with the addition of 3% nano-bentonite content stabilizes. Observations suggest that the addition of nano-bentonite improves the resistance of clayey sand acting as a binder. The results on Fig. 10 show the influence of curing period on the secant modulus at 50% of peak stress with 3% of additive of the micro-bentonite and the nano-bentonite.

On 1d, the secant modulus at 50% of peak stress (E50) of micro-bentonite and nano-bentonite are 2500 and 2000 kg/cm^2^ respectively. On 7d, the secant modulus at 50% of peak stress of micro-bentonite and nano-bentonite are 2300 and 4500 kg/cm^2^ respectively. A reduction in the secant modulus at 50% of peak stress are observed for micro-bentonite while that of nano-bentonite doubles. On 28d, the E50 of micro-bentonite and nano- bentonite are 2300 and 6000 kg/cm^2^ respectively. A further decrease in the secant modulus at 50% of peak stress is observed for the micro-bentonite while that of the nano-bentonite continues to increase. A decrease in secant modulus at 50% of peak stress over time is observed for micro-bentonite. However, a strong increase in secant modulus at 50% of peak stress over time is observed for nano-bentonite. On 1d, the E50 is 2000 kg/cm^2^ and approaching 6000 kg/cm^2^ on 28 d. The E50 is thus 3 times higher on day 28 than on 1d. Observations suggest that nano-bentonite with 3% additive has higher secant modulus at 50% of peak stress over time, whereas that of micro-bentonite degrades.

**Figure 11:**
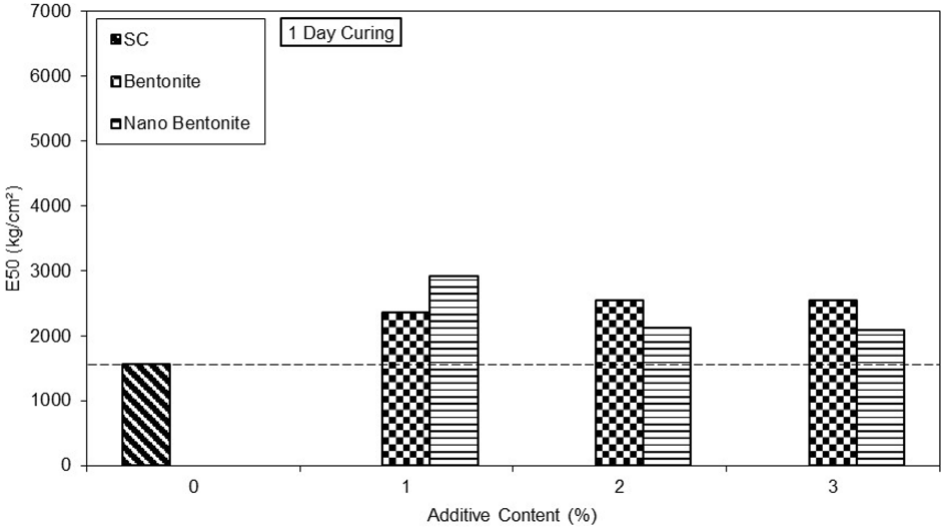
E50 versus untreated and treated clayey sand specimens with various percentages of micro- and nano- bentonite in 1 day curing time.

The results on the bar graph in Fig. 11 show the additive content as a function of the secant modulus at 50% of peak stress of clayey sandy soil, micro-bentonite and nano-bentonite at 1 day of curing time.

The clayey sand without additives achieves E50 secant modulus at 50% of 1500 kg/cm^2^. With the addition of 1% of micro-bentonite and nano-bentonite it reaches respectively 2500 and 3000 kg/cm^2^ at one day of curing time.

The E50 modulus of the soil treated with micro-bentonite and nano-bentonite is higher than that of clay sand specimen.

### SEM

**Figure 12:**
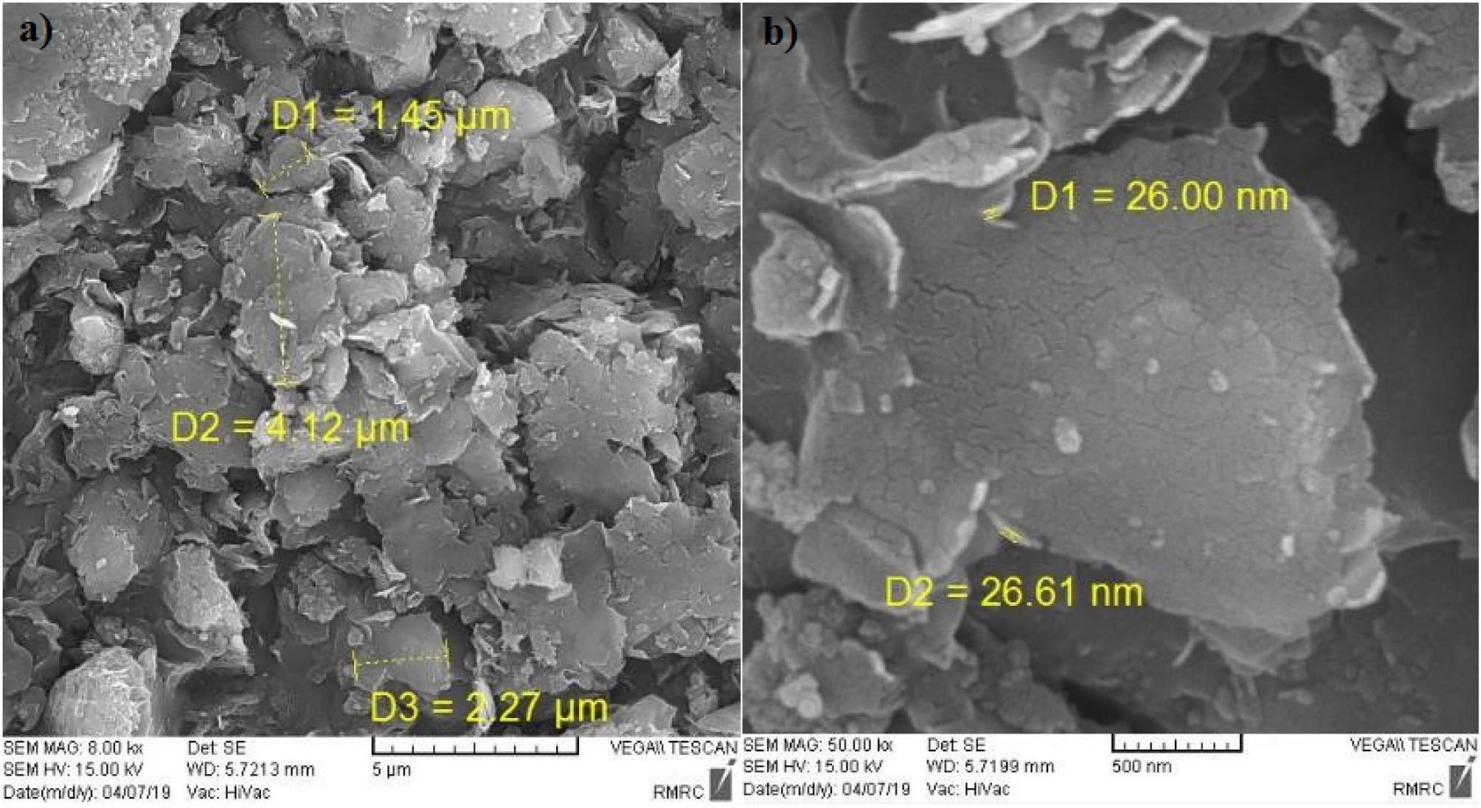
SEM micrographes of (**a**) treated clayey sand sample with micro-bentonite additives, (**b**) untreated soil sample for 1d curing times.

**Figure 13:**
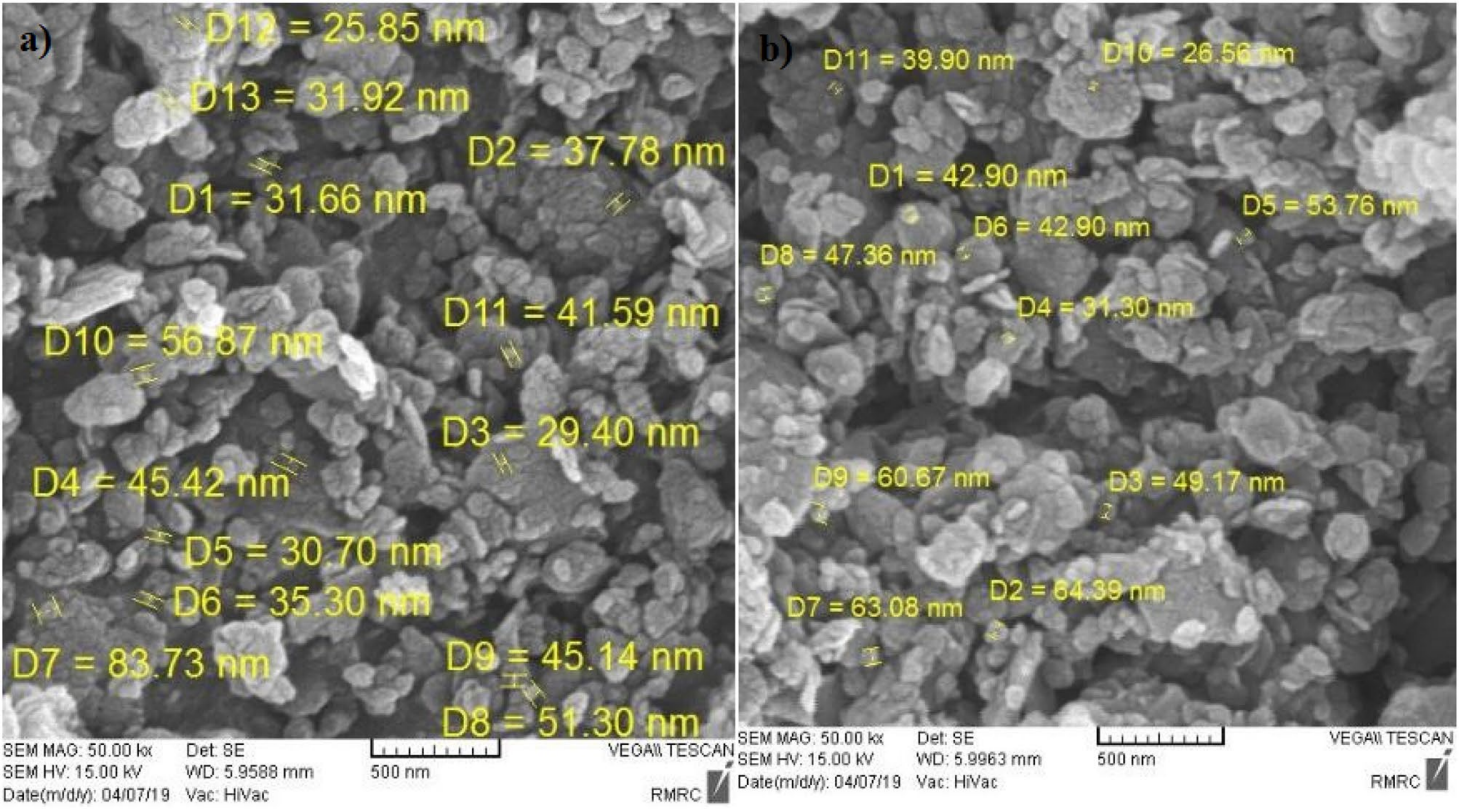
SEM micrographes of (**a**) treated clayey sand sample with nano-bentonite additives for 28d curing time, (**b**) treated clayey sand sample with nano-bentonite additives for 7d curing times.

Examining the morphology of clayey sand specimens with micro- and nano- bentonite, Scanning Electron Microscopy (SEM) showed the cementation mechanism that leads to improving the mechanical properties of soil samples. SEM involves using an electron beam to scan the surface of a sample and produce its two-dimensional image. Untreated and treated soils have very complex structure where it is not easy to demonstrate the presence of micro- and nano-particles using SEM although micro- and nano- bentonites were observe in various SEM images shown in Figs. 12 and 13. Various micro-bentonites type structures, were seen with micro-size scale in different percentages 0, 1, 2, and 3% in specimens, shown in Fig. 12a. The morphology of untreated clayey sand sample is observed in Fig. 12b. High-resolution observation showed that micro-bentonite particles are arranged in layers and have a sheet-like shape, which could be explained by their ability to form an aggregated particle structure. The observed particles have relatively irregular edges and a rough surface with asperities of different sizes. In conclusion, micro-bentonite has a sheet shape which could account for its ability to form a layer and clump together.

Scanning electron microscope analysis of the nano-bentonite particles in Fig. 13 revealed a uniform and relatively small particle aggregate structure. The nano-bentonite (NB) additive increased particle aggregation with the gel formation holding the soil particles together that it is cause of higher reactivity of nano-bentonite to occur with clayey sand particles. High-resolution observation showed that the nano-bentonite particles have a spherical shape, with a smooth and irregular surface. This spherical shape and smooth surface could explain the dispersion stability of nano-bentonite in aqueous solutions. In addition, nano-bentonite has higher porosity and permeability than traditional bentonite. This could increase its effectiveness as an additive to improve clayey sand soils.

In order to confirm the nano-bentonite additive reaches the maximum strength improvement in clayey sand specimens including 3% nano-bentonite, the samples were evaluated with SEM. SEM images provided good observation about the nano-bentonite process and change of surface compositions in different clayey sand soil samples.

### XRD

**Figure 14:**
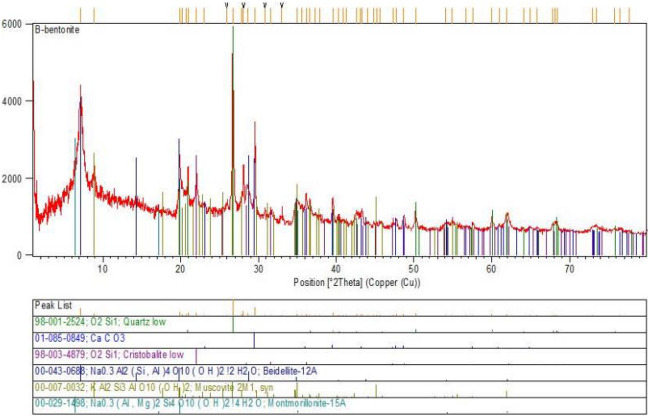
XRD pattern of treated clayey sand specimen with micro-bentonite.

X-ray diffraction (XRD) testing is a method used to study the crystal structure of a substance. In order to confirm the nano-bentonite additive reaches the maximum improvement in clayey sand specimen including 3% nano-bentonite, samples were evaluated with XRD. The results showed that micro-bentonite is characterized by a very fine and homogeneous crystalline structure. The diffraction peaks observed on the graph of Fig. 14 indicate the major presence of Quartz and Muscovite which are responsible for the characteristic smectite structure of bentonite.

In general, the results of the X-ray diffraction (XRD) test confirm that micro-bentonite is indeed a smectite clay and show that the crystal structure of bentonite is very fine and homogeneous. This kind of crystal structure is very favorable for geotechnical applications, as it can improve the shear strength and stability of soft soils. Figure 14 shows the XRD pattern of treated clayey sand soils with micro-bentonite. In conclusion, the results of the X-ray diffraction test for micro-bentonite are very promising for geotechnical applications and suggest that micro-bentonite can be used effectively to improve the properties of clayey sand.

**Figure 15:**
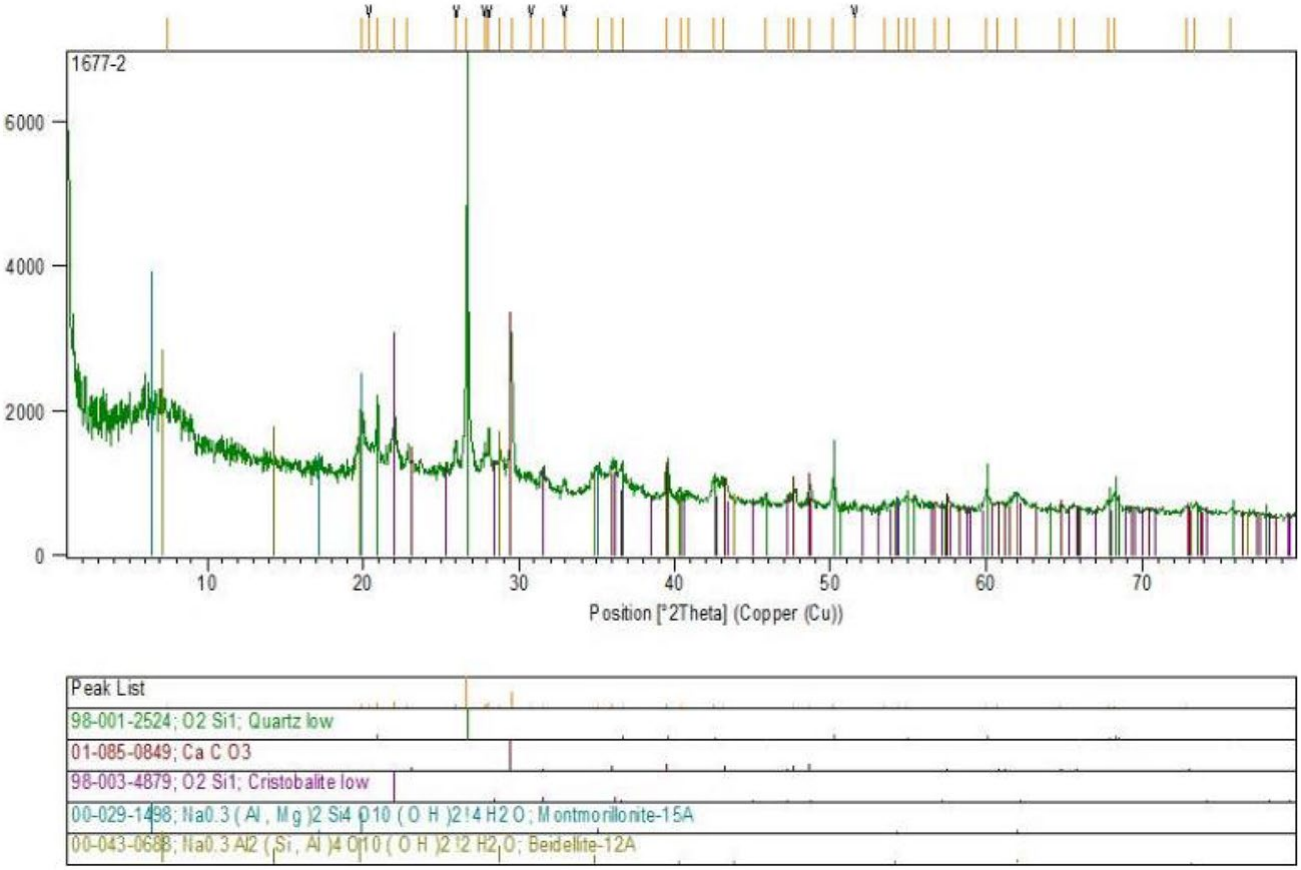
XRD pattern of treated clayey sand with addition of nano-bentonite.

Observing the results of the X-ray diffraction test for nano-bentonite shows distinct and well-defined peaks at specific diffraction angles. The diffraction peaks on the graph in Fig. 15 correspond to specific distances between the atoms of the nano-bentonite, indicating a well-defined and organized crystal structure.

The results of this test show that nano-bentonite has a different crystal structure than conventional micro-bentonite, which may explain the differences in the properties of these two materials. Indeed, nano-bentonite is mostly composed of Quartz and has a larger surface area, which can lead to improved bonding properties when used to improve clayey sand.

In order to confirm the nano-bentonite additive reaches the maximum strength improvement in clayey sand specimen including 3% nano-bentonite, XRD tests were performed. In conclusion, the results of the X-ray diffraction test shows that nano-bentonite has a well-defined crystal structure different from that of conventional micro-bentonite, which may explain the differences in their properties. The observations from this test are very important for the improvement of clayey sand, as they show that nano-bentonite can be a promising material for this application due to its unique properties.

## Conclusions

According to the literature review, most researchers used micro bentonite in soil improvement; also, they conducted the powder method for soil stabilization. This research concluded that micro-bentonite can be a viable option for soil stabilization, but it requires more research and optimization to determine the optimal conditions and processes for different soils and applications. This research also highlighted the potential benefits of using nano-bentonite in a suspension condition as a novel additive for soil improvement.

This research will promote utilization of micro- and nano-bentonite for soil improvement techniques. This study confirmed the role of micro- and nano- bentonite in nano soil-improvement techniques. In conclusion, our results showed that nano-bentonite can be considered as more effective additive to improve the properties of soft soils compared to micro-bentonite, thanks to its spherical shape, its smooth and uniform surface, its ability to increase soil stability and its higher permeability. These properties could make it superior to traditional bentonite as an additive to improve soft soils. Atterberg limit tests have shown that nano-bentonite additives can reduce plasticity index and improve clayey sand consistency. Our results show that nano-bentonite is more effective over long term in improving the properties of clayey sand than micro-bentonite when combined with 3% content. According to results of this research, the use of 3% nano-bentonite is recommended for significant improvement of untreated clayey sand.

The results showed that nano-bentonite suspension significantly improved the strength and stiffness of the soil-bentonite mixture by forming gel that filled the pores and bonded the soil particles. Nano-bentonite also acted as a nucleation site, enhancing the durability and strength of the mixture. Nano-bentonite suspension improved soil specimens, which filled the voids by the gels. This developed a new method for soil improvement better than the powder method that different researchers used before. Nano-bentonite as a type of nano-additives can be used in soil improvement techniques in clayey sand. This is one of few studies where micro- and nano-bentonite were directly applied in soil stabilization. This research was done in laboratory conditions. Future work is required to do in large scale and field tests.

## Data Availability

The datasets generated during and/or analysed during the current study are available from the corresponding author on reasonable request.
