# Author Correction: Micro- and nano-bentonite to improve the strength of clayey sand as a nano soil-improvement technique

**DOI:** 10.1038/s41598-023-38712-7

**Published:** 2023-07-17

**Authors:** Mohadeseh Cheraghalikhani, Hamed Niroumand, Lech Balachowski

**Affiliations:** 1 Department of Civil Engineering, Faculty of Engineering, Buein Zahra Technical University, Qazvin, Iran; 2 Department of Geotechnical and Hydraulic Engineering, Faculty of Civil and Environmental Engineering, Gdansk University of Technology, Gdansk, Poland

Correction to: *Scientific Reports* https://doi.org/10.1038/s41598-023-37936-x, published online 05 July 2023

In the original version of this Article, Hamed Niroumand was incorrectly affiliated with ‘Department of Geotechnical and Hydraulic Engineering, Faculty of Civil and Environmental Engineering, Gdansk University of Technology, Gdansk, Poland’. The correct affiliation is:

Department of Civil Engineering, Faculty of Engineering, Buein Zahra Technical University, Qazvin, Iran

The original Article has been corrected.

